# Pressure Engineering to Enable Improved Stability and Performance of Metal Halide Perovskite Photovoltaics

**DOI:** 10.3390/molecules30061292

**Published:** 2025-03-13

**Authors:** Erin Burgard, Saivineeth Penukula, Marco Casareto, Nicholas Rolston

**Affiliations:** Renewable Energy Materials and Devices Lab, School of Electrical, Computer, and Energy Engineering (ECEE), Arizona State University, Tempe, AZ 85281, USA; emburgar@asu.edu (E.B.); spenukul@asu.edu (S.P.); mcasaret@asu.edu (M.C.)

**Keywords:** perovskite solar cells, stress, encapsulation, defects, durability, photoluminescence, lamination, reliability, ion migration

## Abstract

In this work, we demonstrate that an external pressure of 15–30 kPa can significantly improve metal halide perovskite (MHP) film thermal stability. We demonstrate this through the application of weight on top of an MHP film during thermal aging in preserving the perovskite phase and the mobile ion concentration, an effect which we hypothesize reduces the extent to which volatile species can escape from the MHP lattice. This method is shown to be effective for a more scalable approach by only applying the weight to a cover glass during the lamination of an epoxy-based resin, after which the weight is removed. The amount of pressure applied during lamination is shown to correlate with stability in both 1 sun illumination and damp heat testing. Lastly, the performance of MHP photovoltaic devices is improved using pressure during lamination, an effect which is attributed to improved interfacial contact between the MHP and the adjacent charge transport layers and healing of any voids or defects that may exist at the buried interface after processing. As such, there are implications for tuning the amount of pressure that is applied during lamination to enable the durability of MHP solar modules toward manufacturing-scale deployment.

## 1. Introduction

Perovskite solar cells (PSCs) offer distinct advantages over conventional silicon (Si)-based solar cells. Their roll-to-roll compatibility makes them cheaper and safer to manufacture, while still achieving power conversion efficiencies (PCE) on par with Si solar cells [1,2,3,4,5]. The greatest impediment to the widespread adoption of PSCs is their instability under environmental conditions such as exposure to moisture, oxygen, heat, and light [6,7,8,9]. Water molecules trigger the decomposition of the chemical structure into similar products as the precursor materials. Oxidation and intense heat or light also cause phase transitions or structural changes. Exposure to continuous or cyclic electric fields, heat, or light may not only disrupt the uniformity but also encourage further degradation by creating defects or vacancies that promote ion migration [10]. Any breakdown of the perovskite structure caused by these variables is detrimental to the material’s optoelectronic functionality, and therefore, it is vital to understand this better and prevent degradation.

Previous work has shown that subjecting PSCs to high pressures (5–7 MPa) using a diamond anvil improved device stability with up to a ~40% relative increase in PCE [11]. With lower, more reasonable pressures of 40–50 kPa, a 7% increase in relative PCE was achieved [12]. By applying pressure, the device showed better interlayer contact and reduced interatomic distances. Enhanced contact between the layers is especially desirable because it restrains crack growth along the interfaces and allows for better charge conduction. Compressing the lattice structure thus has the potential to increase the activation energy for ion movement and limit structural decomposition in MHPs. High external pressure was also found to increase the range of stable halide mixing ratios and modify the bond lengths and valence angles, therefore creating a more useful solar material [13,14,15]. However, the effects reverse once the pressure is released. Fortunately, the encapsulation processes in the post-fabrication process inherently incorporate pressure that can be used to this advantage.

The pressure used during typical laminated polymeric encapsulants is on the order of ~100 kPa [16]; there is an opportunity essential to preserve the benefits of pressure-induced properties after the pressure is no longer applied. The highly deformable, thick encapsulant layers are commonly materials such as ethylene vinyl acetate (EVA) or polyolefin [17], which can plastically deform and dissipate the applied pressure to some extent. As such, pressure is applied during module encapsulation to ensure proper adhesion rather than for the purpose of enhancing the electronic properties of the device. Additionally, the familiar encapsulation and pressure methods used for silicon solar cell manufacturing require modification for PSCs since they have a limited thermal budget [18,19,20]. For instance, upon initial consideration, while EVA is commonly used for silicon solar cells, it is largely incompatible with PSCs due to its potential to release acetic acid, which can decompose the MHP [21]. Polyolefin elastomers and thermoplastic polyurethane are more commonly used [21,22], and processing conditions have been optimized to minimize thermal exposure during lamination in recent work. Although pressure is used for lamination in all of these cases, the effect that pressure has independent of the use of packaging materials is not well understood. As such, there remains a valuable opportunity to improve device stability and performance by decoupling the effects of pressure from the encapsulation process.

In this work, we used pressures of 15.2 and 30.4 kPa during the encapsulation of MHP thin films using epoxy resin and PSCs using UV-curable resin to improve stability. As a thermosetting polymer, the epoxy hardened under applied pressure and maintained the induced pressure after the weight was removed. Our findings demonstrate that encapsulation along with the application of pressure enhances the stability of MHP films and the performance of PSCs.

## 2. Results

### 2.1. Continuous Pressure to Simulate Encapsulation

**Figure 1:**
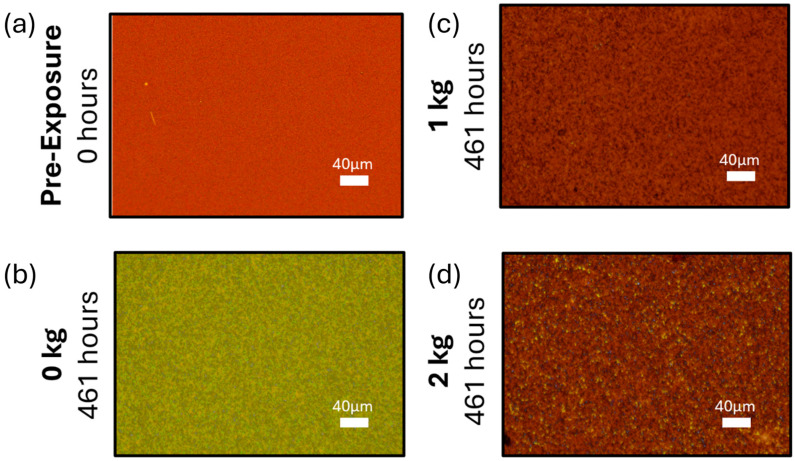
Optical microscope images of (**a**) control MHP film before exposure to heat, (**b**–**d**) MHP films after exposure to heat at 85 °C, 0% humidity for 461 h (**b**) with no pressure on top, (**c**) with 15.2 kPa pressure (**d**) with 30.4 kPa pressure.

An initial pressure test was performed by placing weights of 1 kg (15.2 kPa) or 2 kg (30.4 kPa) on MHP films continuously and compared against control samples with no weight applied. Accelerated aging was performed on the MHP films under applied pressure by exposing them to 85 °C heat in an N_2_ glovebox. Transmission optical images were collected pre-exposure and post-exposure (Figure 1) to observe any morphological changes in the films with aging. After aging, the control MHP film with no applied pressure on top of it turned yellow (Figure 1b), indicating degradation and loss of the perovskite phase. By comparison, the MHP film with 15.2 kPa pressure on top of it did not exhibit any major changes when compared to the pre-exposure image (Figure 1c). Surprisingly, the MHP film with 30.4 kPa pressure on top of it had the appearance of visible yellow spots, suggesting that the higher amount of pressure for such a long time may have led to local degradation of the film (Figure 1d).

**Figure 2:**
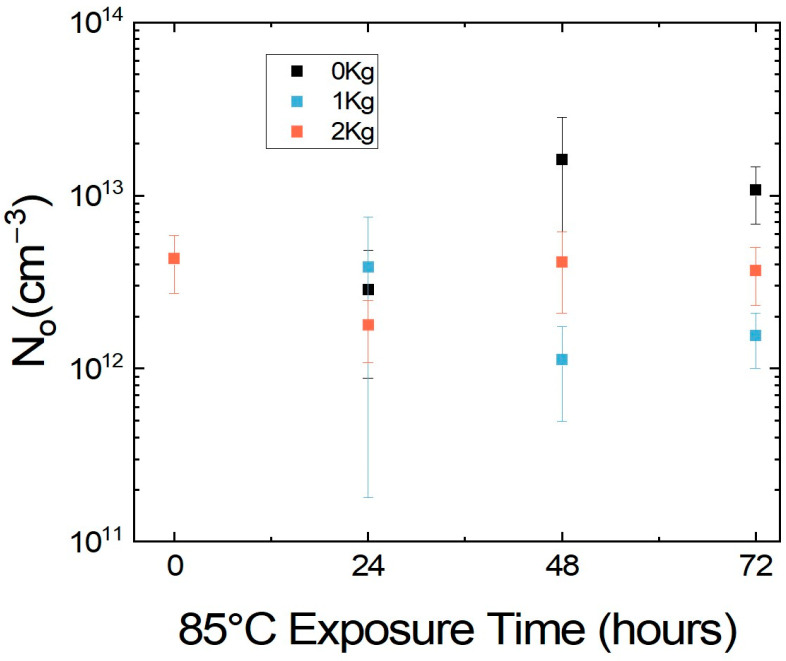
N_o_ vs. exposure time for MHPs tested with a pressure of either 0 kPa, 15.2 kPa, or 30.4 kPa applied on top of them while aging at 85 °C for a total period of 72 h. N_o_ (cm^−3^) is a density measurement, referring to the quantity of mobile ions per cubic centimeter in the material.

To better understand the mechanism for this change in stability with external pressure, a carbon top electrode was applied to MHP films on ITO that were subjected to a temperature of 85 °C in an N_2_-glovebox for 72 h, with a pressure of either 0 kPa, 15.2 kPa, or 30.4 kPa applied on top of the MHPs, and mobile ion concentration (N_o_) measurements were performed on MHPs periodically (Figure 2). The MHP without pressure showed a significant change in N_o_ with almost an order of magnitude increase from 4.3 × 10^12^ cm^−3^ to 1.0 × 10^13^ cm^−3^ after aging for 72 h, indicating an increase in mobile halide ions activated from heat. This is in line with previous measurements we have reported for mobile ion concentration at elevated temperature [23]. By comparison, the MHP with 15.2 kPa pressure exhibited a minor reduction in N_o_ from 4.3 × 10^12^ cm^−3^ to 1.5 × 10^12^ cm^−3^ after aging for 72 h. However, the MHP with 30.4 kPa pressure almost retained the initial N_o_ after aging for 72 h with a negligible change from 4.3 × 10^12^ cm^−3^ to 3.6 × 10^12^ cm^−3^. Note that any increase in N_o_ is not desirable as it indicates either an increase in ion migration or an eventual degradation. Hence, these changes in N_o_ with time show that having pressure on top of MHP markedly improved thermal stability both visually and by reducing ion migration.

### 2.2. Tuning Pressure During Encapsulation of MHP Films and Devices

**Figure 3:**
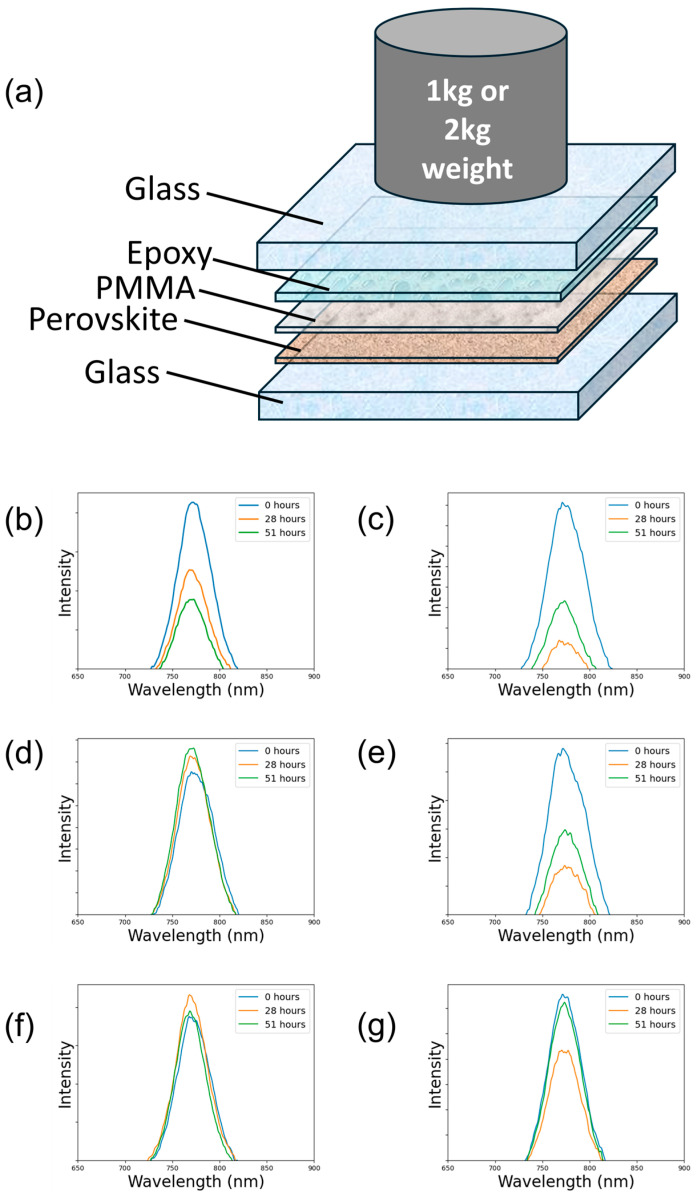
(**a**) Schematic of device layering during the curing process of the epoxy encapsulant, PL data with light exposure (51 h of continuous solar operation at 1000 W/m^2^ (1 sun)) of encapsulated MHP samples under a pressure of (**b**) 0 kPa, (**c**) 15.2 kPa, and (**d**) 30.4 kPa. PL data with damp heat exposure (185 h of damp heating at 85 °C/85% relative humidity) of encapsulated MHP samples under a pressure of (**e**) 0 kPa, (**f**) 15.2 kPa, and (**g**) 30.4 kPa.

The prospect of applying a weight continuously on an MHP film is not realistic. As such, a subsequent set of experiments were performed to subject the MHPs to either no pressure (0 kPa) or a pressure of 15.2 kPa or 30.4 kPa during the encapsulation process. A two-part cross-linking epoxy cured at room temperature was applied on top, and a polymethyl methacrylate (PMMA) layer was deposited above the MHP to prevent any chemical reaction between the epoxy and the MHP (Figure 3a). The weights were placed above the device for the duration of the curing process and removed after 24 h upon which the epoxy was assumed to be fully cross-linked. The encapsulated samples were then exposed to accelerated aging conditions and characterized using photoluminescence (PL) measurements periodically.

The amount of pressure during encapsulation significantly affected degradation induced by both continuous 1 sunlight (full spectrum Xenon arc lamp in ambient) and damp heat (85 °C, 85% RH) exposure. Samples produced without pressure exhibited a progressive decline in PL, indicating a sustained loss of device stability over time under environmental conditions. In contrast, samples produced with a pressure applied (15.2 kPa and 30.4 kPa) demonstrated a remarkably lower, or nonexistent, reduction in PL throughout the same 51 h exposure period (Figure S1). In the illumination exposure test, the sample cured with no pressure exhibited a 58% reduction in the PL peak (Figure 3b), while the samples cured with an applied pressure of 15.2 kPa and 30.4 kPa exhibited a 17% (Figure 3c) and 3.9% (Figure 3d) increase in the PL peak, respectively. Likewise, the damp heat exposure yielded a 59% (Figure 3e), 49% (Figure 3f), and 4.8% (Figure 3g) reduction in their PL peaks for 0, 15.2, and 30.4 kPa pressures, respectively. It is evident that the weighted epoxy curing process reduces degradation in both variable exposure tests. This suggests that the application of pressure during the epoxy encapsulation curing process potentially promotes an enhanced interface even after the pressure is removed, which we attribute to the effects of sustained pressure improving the MHP film quality and interfacial contact. We note that this effect is possible because a brittle epoxy is unlikely to absorb or dissipate pressure in the same manner as a laminated polymeric encapsulant. There is, however, also the possibility of a denser encapsulant under pressure which would also contribute to the improved stability under damp heat. It is clear that pressure minimizes with these experiments, but continued studies with pressurized encapsulation should be performed to determine the ideal amount of pressure to be added during the curing process to ensure lasting stability.

This experiment substantiates two key concepts: (1) the pressure applied during curing likely persists even after the removal of the weight, and (2) this pressure contributes to the enhanced optical and PL stability of the samples. Since there is a different result between weighted and unweighted samples, the brittleness and relative thinness (~10 µm) of the epoxy during the curing process suggests that the pressure could be retained after the weight is removed.

Finally, to examine the effects of pressure on device performance and to further enable manufacturability by reducing pressure time from 24 h to 1 min, PSCs with a p-i-n device structure were fabricated ITO/NiO_x_/1.2 M Cs_0.2_FA_0.8_PbI_3_ MHP/C_60_/Ag. Devices were encapsulated using pressure and a UV-curable resin. In this case, a pressure of 30.4 kPa was applied for 1 min to the top cover glass to effectively spread out the resin, after which the pressure was removed and the device placed under a UV lamp to cure for 5 min. As shown in Figure S2a, an immediate sharp increase in PCE was observed. The JV curves shown in Figure S2b demonstrate that the most significant changes in device performance are due to an increase in fill factor and V_oc_, suggestive of improved interfacial contact and better charge transport within the PSC. We believe that a potential reason for this drastic improvement in PCE is primarily due to the compression of the device stack from the 30.4 kPa of pressure inducing a healing effect on any voids or gaps throughout the device structure, such as those seen in Figure S3. This also explains the lower initial PCE values, as we hypothesize that the antisolvent process is not always producing optimal quality, fully dense MHP films. Additional JV measurements and PCE comparisons of devices that underwent solely a 30.4 kPa press for 1 min or UV light exposure for 5 min were also made, which can be seen in Figure S4. Devices subjected solely to pressure and UV light utilized an identical cover glass to those used in encapsulation. There was a slight increase in performance from applying pressure and a slight decrease in performance from UV light exposure.

Applying pressure without encapsulation and then fully removing the pressure for subsequent characterization could undo the effects of better interfacial contact and void healing to produce less of an increase in performance. It is worth noting that the curing time for the UV-resin was mistakenly thought to be 5 min at the selected irradiance when only ~35 s is necessary given the intensity of the UV lamp used. We believe that the slight decrease in performance of some samples exposed just to UV light is indicative of too long of an exposure time without the UV-curable resin in place to absorb most of the UV light, leading to some slight degradation of the MHP and a slightly lower short-circuit current density.

## 3. Methods

The cleaning of the substrate before deposition of MHP is performed as follows: silica glass or indium-doped tin oxide coated glass (ITO-glass) is cleaned in an ultrasonic cleaner with Extran soap solution. ITO-glass is further cleaned with DI water, acetone, and IPA for 10 min each before subjecting them to UV-ozone treatment for 10 min.

### 3.1. Fabrication

#### 3.1.1. Methylammonium Lead Iodide (MAPbI_3_)

The precursor solution for methylammonium lead iodide was prepared by mixing methylammonium iodide (MAI) (Greatcell Solar Materials, Queanbeyan, Australia) and lead iodide (PbI_2_) (TCI America (Montgomeryville, PA, USA)–99.99% trace metal basis). A 1 M concentration solution was made by mixing 0.159 gm of MAI and 0.461 gm of PbI_2_ in a solvent of 4:1 dimethylformamide (DMF) (Sigma-Aldrich (St. Louis, MO, USA)–Anhydrous 99.8%) to dimethyl sulfoxide (DMSO) (Sigma-Aldrich–Anhydrous ≥ 99.9%) with 800 µL DMF and 200 µL DMSO. The solution was mixed in a vortex mixer until a fully dissolved solution was formed. MAPbI_3_ films on ITO-glass were fabricated following the same procedure showcased in previous work [24].

#### 3.1.2. NiO_x_

Nickel oxide (NiO_x_) (99.999% trace metal basis) sol-gel was prepared by mixing 1.454 g of nickel nitrate hexahydrate with 4.7 mL of ethylene glycol (EG) (Thermo scientific (Waltham, MA, USA)–anhydrous 99.8%) and 0.3 mL of ethylenediamine (EDA) (Thermo scientific–99%) in a 96:4 volume ratio to create 5 mL of 1 M NiOx sol-gel. The ethylene glycol was added to the nickel nitrate hexahydrate precursor powder before the addition of the ethylenediamine. The sol-gel was left to shake in a vortex mixer for 120 min and filtered with a 0.22 μm PTFE filter before spin-coating.

#### 3.1.3. Cesium Formamidinium Lead Iodide (Cs_0.2_FA_0.8_PbI_3_)

The perovskite used was 1.2 M cesium formamidinium lead-iodide with a composition of Cs_0.2_FA_0.8_PbI_3_ and was prepared in the following manner: 0.1248 g of Cesium Iodide (CsI) (Sigma-Aldrich–99.999% trace metal basis), 0.33 g of Formamidinium Iodide (FAI) (Greatcell Solar Materials), and 1.1064 g of PbI_2_ were weighed out and placed in a vial. Amounts of 1.6 mL of DMF and 0.4 mL of DMSO were added in a 4:1 volume ratio to make 2 mL of 1.2 M Cs_0.2_FA_0.8_PbI_3_. The solution was left to shake in a vortex mixer for 90 min and filtered with a 0.22 μm PTFE filter before spin coating.

#### 3.1.4. PSC Processing

PSCs with p-i-n architecture were fabricated on ITO-glass slides that were cleaned and surface-treated as described previously. The NiO_x_ was spin-coated at 5000 rpm for 30 s with a 2500 rpm ramp-up, and the samples were then annealed at 315 °C for 60 min. The perovskite film was deposited by placing 100 μL of precursor solution onto the substrate and spinning in a two-step method: 500 rpm for 10 s with a 250 rpm ramp-up followed by 3500 rpm for 20 s with a 1000 rpm ramp-up. An amount of 400 μL of chlorobenzene was deposited 10 s into the second step as an antisolvent. The samples were then annealed at 120 °C for 15 min. Amounts of 40 nm of C60, 8 nm of bathocuproine (BCP), and 100 nm of silver were all thermally evaporated in an Angstrom thermal evaporation chamber located inside an N_2_-filled glovebox to serve as the electron transport layer, hole-blocking/buffer layer, and back electrode, respectively. Contact with the ITO layer was made by scraping off all device layers on one edge of the substrate and applying a thin layer of conductive silver paint (Ted Pella, Redding, CA, USA) which was left to dry in N_2_ for one hour. Devices were then taken for testing and encapsulation.

#### 3.1.5. Encapsulation Approach

A UV-curable resin acquired from Everlight Chemical USA (Pineville, NC, USA) was used as the encapsulant (ID# AB-302). A drop of the resin was placed on a cleaned cover glass and spread with a wooden applicator stick. The cover glass was then placed over the active area of PSC and held in place for 1 min with a 2 kg weight (30.4 kPa). After 1 min, the weight was removed and the sample was immediately placed underneath a UV lamp with a wavelength of 365 nm and intensity of 1.25 W∙cm^−2^ for 5 min to cure the resin.

### 3.2. Pressure Application Approach

Standard weights of 1 kg (15.2 kPa) and 2 kg (30.4 kPa) were used to induce pressure on MHP by placing the weights on top of the MHP substrates. This approach is called the “pure” pressure approach where there is no other layer between MHP and the weight. It served as the initial test to validate the preliminary concept of minimizing degradation with pressure.

The following “induced” pressure approach involved the encapsulation of MHP and applying pressure on the MHP during the encapsulation process. The MHP was first covered with a protective coating of poly methyl methacrylate (PMMA) before coating epoxy (Epotek 301, Epoxy Technology, Billerica, MA, USA) on it and encapsulating MHP with a top glass. After applying the epoxy and top glass, the samples were set to cure in an N_2_ glovebox with either no weight, 1 kg (15.2 kPa), or 2 kg (30.4 kPa) weights placed on top of the encapsulated samples. After 24 h, the weights were removed.

### 3.3. Characterization

A hot plate was used to expose the MHPs to heat in an N_2_ glovebox at 85 °C for 461 h. A Thermotron environmental chamber was used to expose the MHPs to a relative humidity of 85% at 85 °C for 51 h. A Xenon arc lamp (Asahi Spectra, Torrance, CA, USA) at 1 sun intensity was used to expose the MHPs to light for 51 h.

The morphology changes in the samples were observed using an optical microscope (Keyence, Itasca, IL, USA), and photoluminescence (PL) was measured using an in-house BLACK-Comet UV–Vis Spectrometer from StellarNet (Tampa, FL, USA) with a laser wavelength of 425 nm. The microscope images, taken at 0 h and 461 h of heat exposure, were used to visually validate the continuation of our experiment as the color change from dark red to yellow represents an unwanted phase transition due to ion migration. PL analysis was chosen to characterize the samples because degradation and ion migration shift the PSCs radiative recombination to non-radiative combination, causing a clear decrease in the PL peak. Measurements were taken after 28 h; then, the samples were placed back in the heat or light environments for continued testing until 51 h. The evolution of ionic properties of the MHPs was measured using PAIOS (Characterization suite version 4.4.1), an all-in-one characterization tool for photovoltaic devices and LEDs. The method for measuring and calculating N_o_ was used as described in our previous work [24]. While measuring N_o_, all the MHPs were measured at 0 h as is without placing a weight on top of them. Glass substrates were then placed on top of the MHPs before exposing them to heat following the observations in previous work to simulate a device like architecture and to prevent the loss of iodine in gaseous form [23]. Weights of 0 kg, 1 kg (15.2 kPa), and 2 kg (30.4 kPa) were placed on top of the samples while they were being exposed to heat of 85 °C, and they were measured periodically every 24 h for a period of 72 h.

### 3.4. Device Characterization

Devices were characterized using an Oriel Sol3A solar simulator with a Xenon lamp at a 1-sun intensity and a spectrum of AM1.5 G (Newport Corporation, Irvine, CA, USA). Current–voltage sweeps were performed in reverse bias from 1.2 V to −0.2 V with a voltage step-size of 0.014 V and dwell time of 10 ms.

## 4. Conclusions

Ionic and operational (thermal and light) stability was improved in MHP films from applied pressure. This external pressure may be retained with weighted epoxy curing in the encapsulation process. The device performance of PSCs also improves with reduced series resistance, attributed to better interface contact between the MHP and charge transport layers. Future work will quantify the effect of pressure on changes to the strain level/film stress of the MHP layer and explore tuning the MHP process parameters to reduce the number of voids initially present in the film to increase baseline PSC performance. Our results suggest that the amount of pressure applied directly to the perovskite is an important variable for yielding desired improvements in stability.

## Data Availability

The original contributions presented in this study are included in the article/Supplementary Material. Further inquiries can be directed to the corresponding author.

## Supplementary Materials

The following supporting information can be downloaded at: https://www.mdpi.com/article/10.3390/molecules30061292/s1. Figure S1: Tables showing PL data after continuous 1-Sun light exposure (for 51 h) for encapsulated MHP samples under a pressure of (a) 0 kPa, (b) 15.2 kPa, and (c) 30.4 kPa. Tables showing PL data after damp heat exposure (85 °C, 85% RH) (for 51 h) for encapsulated MHP samples under a pressure of (d) 0 kPa, (e) 15.2 kPa, and (f) 30.4 kPa. Figure S2: (a) Enhancement of power conversion efficiency (PCE) of devices after undergoing the encapsulation process and (b) a representative comparison of JV-curves of a device before and after encapsulation. Figure S3: SEM image of Cs_0.2_FA_0.8_PbI_3_ film made using the antisolvent method. Figure S4: JV-sweeps of devices that have undergone (a) 30.4 kPa pressure for 1 min and (b) UV light exposure for 5 min and (c,d) their respective PCE statistical plots.
