# Supplementary material for: Pressure Engineering to Enable Improved Stability and Performance of Metal Halide Perovskite Photovoltaics
Source: Molecules. 2025 Mar 13;30(6):1292. doi: 10.3390/molecules30061292 (PMC11944748; doi:10.3390/molecules30061292)
Supplement: Supplementary file 1 [file molecules-30-01292-s001.zip › molecules-3516352-supplementary.pdf]

Pressure Engineering to Enable Improved Stability and Performance of Metal Halide Perovskite Photovoltaics

Erin Burgard, Saivineeth Penukula, Marco Casareto and Nicholas Rolston *

Renewable Energy Materials and Devices Lab, School of Electrical, Computer, and Energy Engineering (ECEE), Arizona State University, Tempe, AZ 85281, USA; emburgar@asu.edu (E.B.); spenukul@asu.edu (S.P.); mcasaret@asu.edu (M.C.)

***** Correspondence: nicholas.rolston@asu.edu

a)

0 kPa		
Time (hrs)	Intensity	
0	4269.8	
28	2533.2	
51	1777.7	
	% Reduction:	58

b)

15.2 kPa		
Time (hrs)	Intensity	
0	3269.1	
28	3632.1	
51	3813.3	
	% Reduction:	-17

c)

30.4 kPa		
Time (hrs)	Intensity	
0	3754.6	
28	4323	
51	3900	
	% Reduction:	-3.9

d)

0 kPa		
Time (hrs)	Intensity	
0	4070.8	
28	691.72	
51	1658.6	
	% Reduction:	59

e)

15.2 kPa		
Time (hrs)	Intensity	
0	2907.2	
28	855.17	
51	1482.3	
	% Reduction:	49

f)

30.4 kPa		
Time (hrs)	Intensity	
0	3275.4	
28	2174.2	
51	3118.1	
	% Reduction:	4.8

Figure S1. Tables showing PL data after continuous 1-Sun light exposure (for 51 hours) for encapsulated MHP samples under a pressure of a) 0 kPa, b) 15.2 kPa, and c) 30.4 kPa. Tables showing PL data after damp heat exposure (85℃, 85% RH) (for 51 hours) for encapsulated MHP samples under a pressure of d) 0 kPa, e) 15.2 kPa, and f) 30.4 kPa.

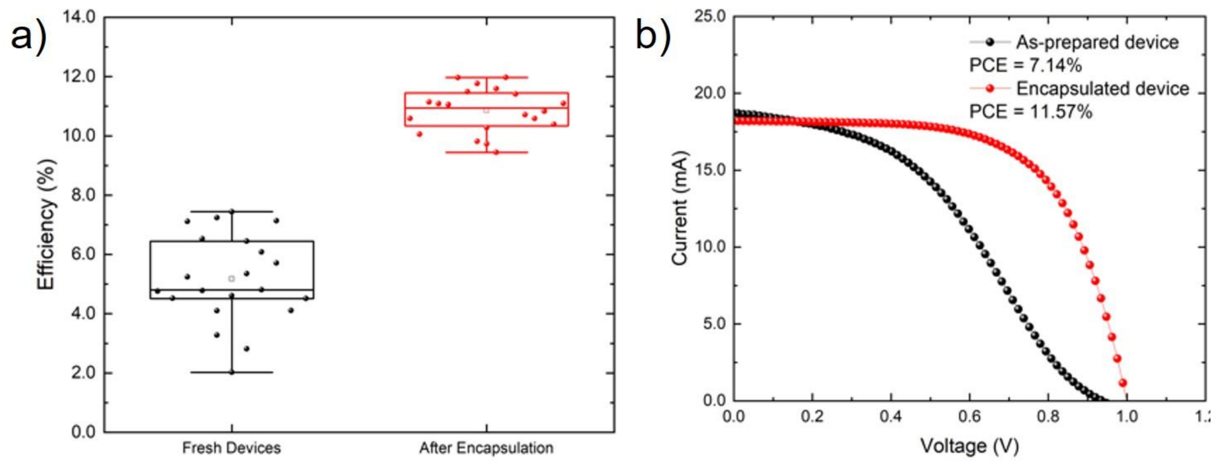

Figure S2. a) Enhancement of power conversion efficiency (PCE) of devices after undergoing the encapsulation process and (b) a representative comparison of JV-curves of a device before and after encapsulation.

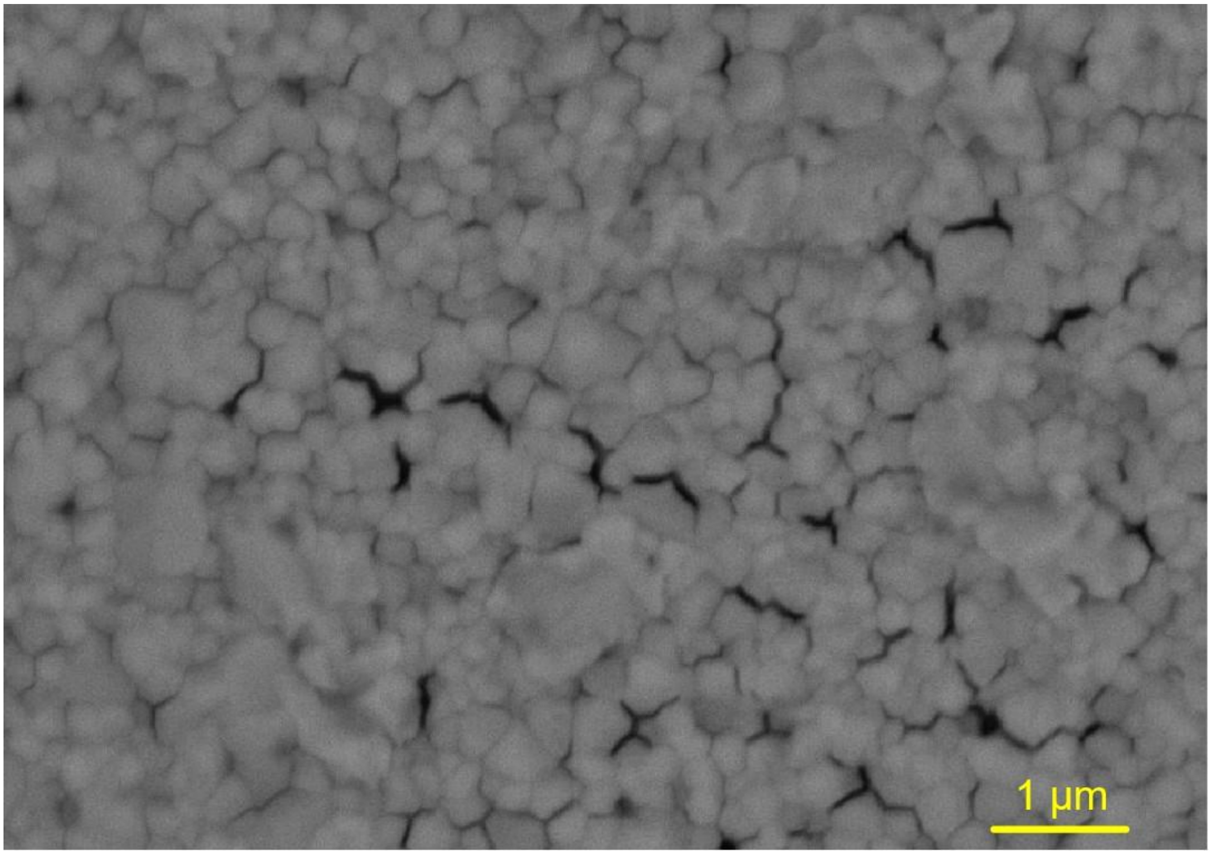

Figure S3. SEM image of $Cs_{0.2}FA_{0.8}PbI_3$ film made using the antisolvent method.

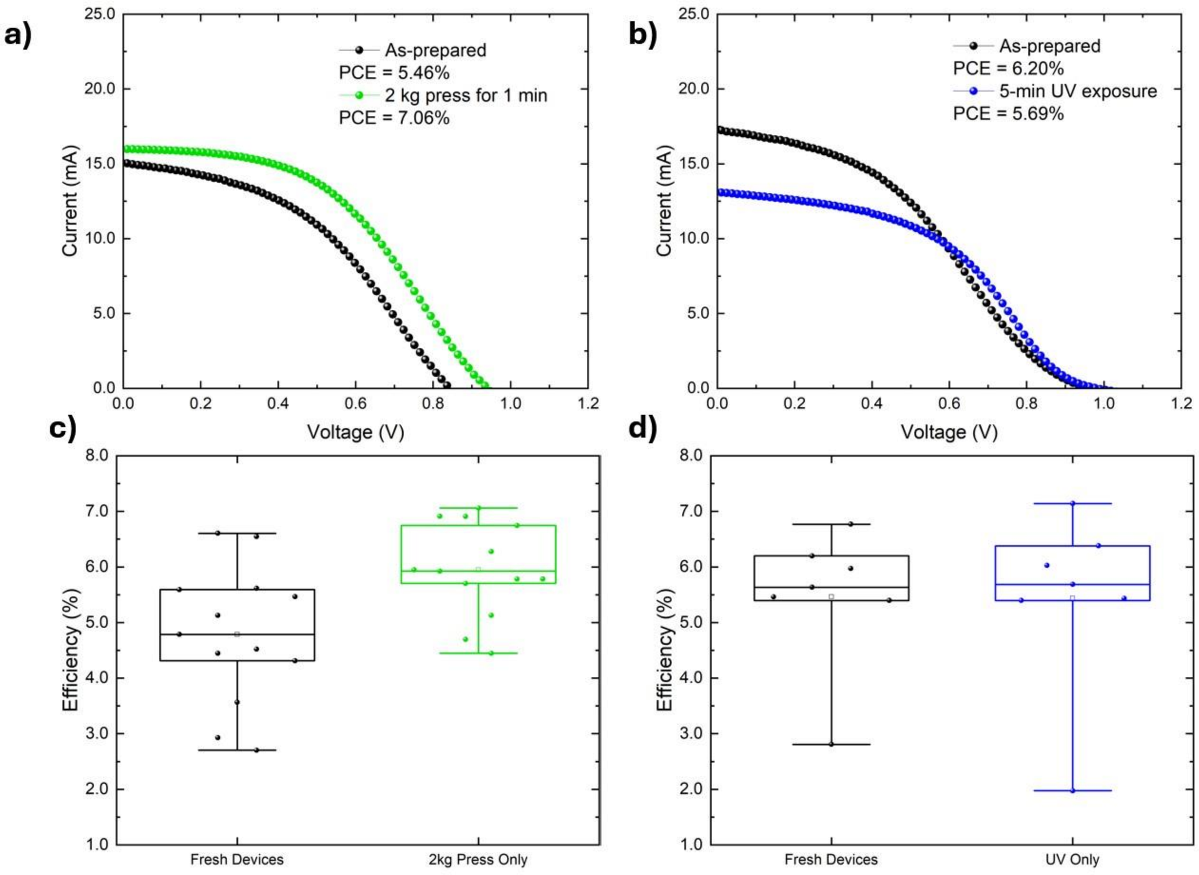

Figure S4. JV-sweeps of devices that have undergone (a) 30.4 kPa pressure for 1 min and (b) UV light exposure for 5 min and (c-d) their respective PCE statistical plots.
